# *Luna*, a *Drosophila* KLF6/KLF7, Is Maternally Required for Synchronized Nuclear and Centrosome Cycles in the Preblastoderm Embryo

**DOI:** 10.1371/journal.pone.0096933

**Published:** 2014-06-10

**Authors:** Ursula Weber, Estefania Rodriguez, John Martignetti, Marek Mlodzik

**Affiliations:** 1 Department of Developmental and Regenerative Biology and Graduate School of Biomedical Sciences, Icahn School of Medicine at Mount Sinai, New York, New York, United States of America; 2 Department of Genetics and Genomic Sciences, Icahn School of Medicine at Mount Sinai, New York, New York, United States of America; University of Massachusetts Medical School, United States of America

## Abstract

Krüppel like factors (KLFs) are conserved transcription factors that have been implicated in many developmental processes including differentiation, organ patterning, or regulation of stem cell pluripotency. We report the generation and analysis of loss-of-function mutants of *Drosophila* Klf6/7, the *luna* gene. We demonstrate that *luna* mutants are associated with very early embryonic defects prior to cellularization at the syncytial stage and cause DNA separation defects during the rapid mitotic cycles resulting in un-coupled DNA and centrosome cycles. These defects manifest themselves, both in animals that are maternally homozygous and heterozygous mutant. Surprisingly, *luna* is only required during the syncytial stages and not later in development, suggesting that the DNA segregation defect is linked to centrosomes, since centrosomes are dispensable for later cell divisions.

## Introduction

Krüppel like factors (KLFs) are highly conserved throughout the animal kingdom and have been implicated in many developmental processes such as differentiation, organ patterning [1], regulation of pluripotency [2], and human diseases [1]. They encode Zinc finger containing transcription factors, which bind DNA and regulate various cellular processes as transcriptional activators or repressors [1]. In evolutionary tree analyses KLF6 clusters with KLF7 [3] and *luna* is a close homolog in *Drosophila* and *Daphnia* [4] [5].

KLF6 is known as a ubiquitously expressed activator associated with proliferation, apoptosis, the hematopoietic system, and various cancers in vertebrates [1]. KLF7, also known as ubiquitously expressed Krüppel like factor (UKLF), is known to regulate sensory neuron development [6] and is involved in fat metabolism [1]. The mouse and zebrafish animal model systems established a KLF6 function in a developing organism [7], [8]. In the mouse, knockout of *Klf6* causes developmental arrest due to failure of erythropoiesis and angiogenesis, and *Klf6^−/−^* embryonic stem (ES) cells show proliferation defects [7]. In zebrafish, morpholino based knockdown revealed that *Klf6/copeb* is essential for the proliferation of endoderm derived tissues [8]. KLF7 knockout mice die shortly after birth due to neuronal defects [6].

In *Drosophila*, early development is characterized by 14 synchronous nuclear divisions in a syncytium, the fertilized egg [9], [10]. The first 9 divisions take place before the onset of zygotic transcription at which point the nuclei migrate to the periphery of the embryo. These processes are solely driven by maternal contribution [9], [11]. De Graeve and colleagues [4] have shown that RNA interference for *luna* aborted development in 50% of the animals prior to gastrulation with large vacuoles forming in the egg yolk and hence coined the gene name *luna*. This approach also affected later developmental stages in *Drosophila* as did over expression of *luna* [4]. However these experiments did not address or reveal for which cellular processes and during which time of development *luna* function was essential.

Here we report the generation of loss-of-function mutants in the *Drosophila luna* gene and show that independent alleles and RNA interference cause the same phenotypic effect. Phenotypic analyses reveal that *luna* function is solely required at early developmental stages during the syncytial divisions, prior to cellularization, and is maternally contributed. Most prominently, *luna* mutants cause DNA separation defects during the early nuclear divisions, while centrosomes proceed their cycling. Hence, we conclude that *luna* is required for the synchronization of nuclear DNA and centrosome cycles.

## Results

### Isolation of *luna* mutants

**Figure 1:**
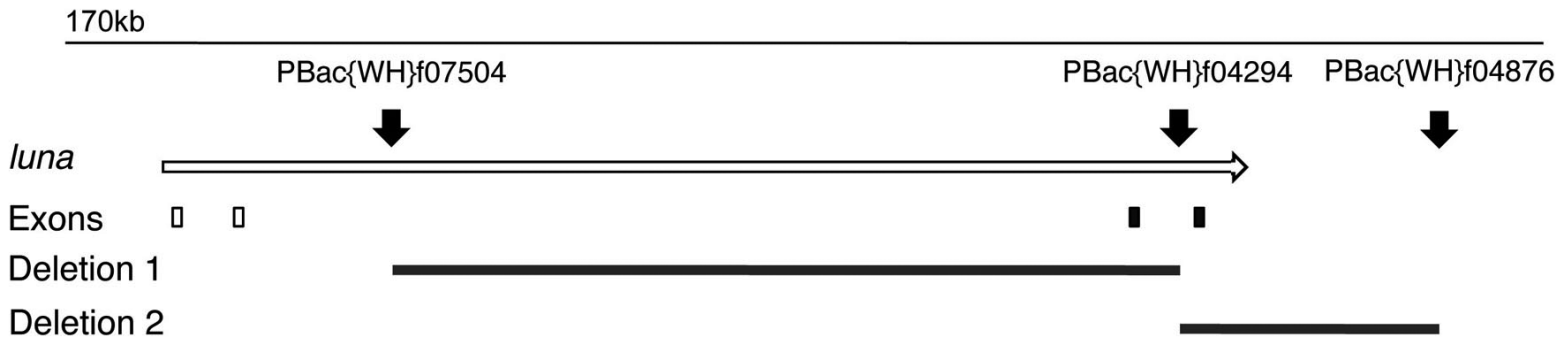
Map of the *luna* locus and mutant alleles. Open arrow indicates the *luna* locus, boxes below show exon containing areas, with non coding or coding sequences, indicated by white or black boxes, respectively. Black arrowheads point to the site and orientation of the PBac insertions used to generate deletion mutants (indicated by black lines).

**Figure 2:**
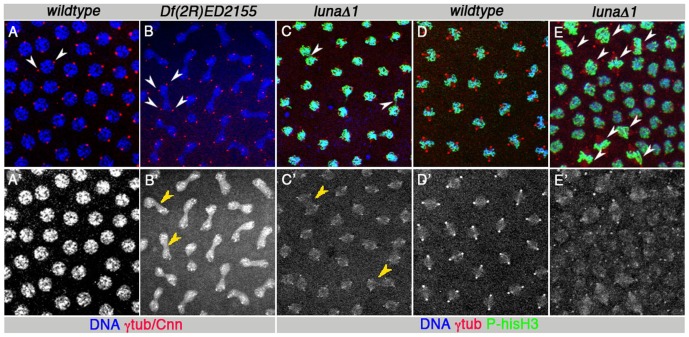
*luna* loss-of-function phenotypes as observed during nuclear division cycles in the early syncytial embryo derived from heterozygous mothers (B, C, E). Control prophase (A) and metaphase (D) “wild-type” embryos from *nos::VP16-GAL4>UAS-white-RNAi/+* mothers, showing normal DNA and division spindle separation and synchrony. All panels show embryos at M10–12 stages when nuclei have reached the embryo cortex with grazing confocal images, representing half an embryo diameter. DNA is stained by Hoechst (blue; in top panels, or monochrome in lower panels A′, B′), centrosomes and spindles are marked by gamma-tubulin (red in A, C–E, monochrome in C′–E′) and centrosomes by anti-Cnn (red in B), and Metaphase condensed DNA is marked with phospho-histone H3 in green (C–E). (B, C) DNA segregation defects are observed in embryos from *luna*-/+ and *Df*/+ mothers and are indicated by yellow arrowheads in B′ and white arrowheads in C. White arrowheads in A, B point to centrosomes. In metaphase, DNA segregation appears coupled with trespassing division spindles indicated by yellow arrowheads in C′. Division cycle asynchrony and possible metaphase arrest phenotypes (E; all DNA is phospho-histone H3 positive). Metaphase stage nuclei in upper left and lower central area indicated by white arrowheads next to smaller, aberrant looking metaphase nuclei in center of panel (unmarked). Maternal genotypes are indicated above each panel. Very similar defects are observed in embryos from heterozygous *luna*Δ*1*, *luna*Δ*2* and *Df(2R)ED2155* mothers, maternally mutant *luna* embryos generated by germline clones and *nos::VP16-GAL4>UAS-luna-RNAi-1* or *-2/+* mothers (see also Figure 3, 4 and Tables 3, 4).

*luna* loss-of-function mutants were generated by combining FLP recombinase and FRT bearing insertions [12], which resulted in two independent, precise genomic deletions, specific to *CG33473/luna*. Each of these removed distinct coding sequences (Figure 1). Due to the fact that all 3 insertions used to generate the gene specific deletions were of the same kind and inserted in the same orientation (Figure 1), standard molecular confirmation of the generated deletions by PCR was not possible, because the recombination generated a 23 kb PBac identical to the two parental ones. Genetic analysis revealed that both alleles were strong loss-of-function mutations, according to their behavior in complementation crosses and phenotypic analyses. No differences of lethal stage or phenotype were observed in embryos from homozygous versus trans-heterozygous *luna* ^−^/deficiency intercrosses (Table 1, Figure 2).

**Table 1:** Complementation analyses.

Parental cross (virgin×male)	F1 CyO (n = )	F1 non Cy (n = )
*luna*Δ*2 (#a29)×luna*Δ*1 (#c7)*	89	0
*luna*Δ*1 (#c10)×luna*Δ*2 (#a23)*	31	0
*luna*Δ*1 (#b5)×Df(2R)ED2155*	12	0
*luna*Δ*1 (#c7)×Df(2R)ED2155*	61	0
*Df(2R)ED2155×FRT42D luna*Δ*1 (#c10)*	250	0
*FRT42D luna*Δ*2 (#a7)×Df(2R)Exel6059*	150	0
*Df(2R)Exel6059×luna*Δ*2 (#a23)*	9	0
*FRT42D luna*Δ*1 (#c7)×Df(2R)ED2155*	250	0

Individually established stocks are indicated by # in parenthesis.

Taken together, we conclude that the two mutant alleles generated are strong loss-of-function alleles.

### *Luna* function is maternally contributed

*luna* lethality manifests itself “zygotic lethal like”. Progeny from *luna* mutant stocks and transheterozygous intercrosses are all heterozygous *luna^−^/CyO* animals (Table 1). Examination of lethal embryos derived from *luna* mutant stocks suggested that *luna* mutants die at preblastoderm stages since 8–20% lethal embryos were found to have arrested development prior to blastoderm stages (Table 2) and CyO homozygous animals were reported to die at 1^st^ instar stages [13]. Similar numbers were obtained from *CyO twi-GFP* balanced *luna* mutant stocks, independent of the paternal genotype (data not shown).

**Table 2:** *luna* mutants manifest themselves as “zygotic lethal like”: determining the lethal stage of *luna* mutants.

*Luna* stock	Total eggs (n = )	Unpatterned embryos	Segmented embryos or larvae
*luna*Δ*1/CyO (#c7)*	265	15.1%	35.8%
*luna*Δ*1/CyO (#b5)*	250	8.4%	31.2%
*luna*Δ*2/CyO (#a7)*	432	20.4%	29.9%
*luna*Δ*2/CyO (#a23)*	123	14.8%	22.8%

Individually established stock are indicated by # in parenthesis.

**Figure 3:**
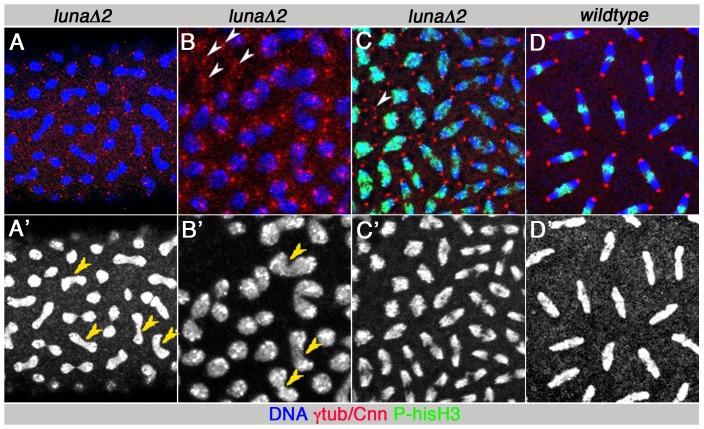
Germline clone derived mutant embryos for *luna*Δ*1* or *luna*Δ*2*. Note same phenotypes as mutant embryos derived from heterozygous mothers. Stainings and stages are as in Figure 2. Lower panels show Hoechst stain. Genotypes of germline clones are indicated above each panel. Compare to control prophase and metaphase embryos in Figure 2A and 2D. DNA segregation defects were seen most prominently during prophase like chromatin stages, with fully segregated centrosomes ready for metaphase (A, B), combined with “nuclear fall out” defects, where centrosomes remain in the periphery and DNA has disappeared (indicated by white arrowheads in B, C). Yellow arrowheads indicate DNA bridges (A′, B′). (C) Asynchronous nuclear division stages combined with nuclear fallout. Upper left corner shows metaphase stage nuclei next to anaphase to telophase stages in the remaining part of panel. Control anaphase stage embryo (D), genotype as controls in Figure 2. Compare Figure 4 and Tables 3,4 for quantitative analysis.

It is unexpected to find such early developmental defects in embryos derived from heterozygous mothers. Furthermore, the zygotic genome is silenced until nuclear cycles 9–10 [14], [15]. We thus examined homozygous mutant eggs (via germline clone technology [16], see Materials and Methods for details) to assess if *luna* function is maternally contributed. Indeed we found that such mutant embryos, lacking the maternal component, died at various early stages of development with up to 20% prior to blastoderm cellularization, possibly due to defects in the nuclear division cycles and similar to those observed in mutant embryos derived from heterozygous mothers (compare Figures 2 and Figure 3, Table 2). Control germline clones did not show such defects (Tables 3 and 4).

**Table 3:** *luna* mutant embryos show severe DNA segregation defects.

Heterozygous mother derived	Maternal genotype	% DNA segregation defect/bridges	n total nuclear figures
	*luna*Δ*1 or 2*	55.4%	56
	*luna*Δ*1 or 2*	46.9%	49
	*luna*Δ*1*	84.8%	198
	*luna*Δ*1 or 2*	19.6%	51
	*luna*Δ*1 or 2*	12.2%	49
	*Df(2R)ED2155*	54.9%	133
	*Df(2R)ED2155*	100.0%	112
	*Df(2R)ED2155*	98.8%	83
	*Df(2R)ED2155*	64.4%	45
	*Df(2R)ED2155*	96.8%	62
	*Df(2R)ED2155*	96.7%	61
	*Df(2R)ED2155*	94.5%	55
	*Df(2R)ED2155*	91.8%	49
	*luna*Δ*1*	100.0%	6
	*luna*Δ*1*	100.0%	5
	*luna*Δ*1*	100.0%	4
	*luna*Δ*1*	88.9%	9
	*luna*Δ*2*	75.7%	70
	*luna*Δ*2*	70.1%	77
	*luna*Δ*2*	75.0%	76
**Germline clone derived**	**Allele**		
	*luna*Δ*1*	40.0%	10
	*luna*Δ*1*	53.3%	15
	*luna*Δ*1*	95.1%	82
	*luna*Δ*1*	32.4%	37
	*luna*Δ*1*	27.5%	40
	*luna*Δ*2*	100.0%	6
	*luna*Δ*2*	60.8%	74
	*luna*Δ*2*	88.9%	45
	*luna*Δ*2*	63.6%	33
	*luna*Δ*2*	23.8%	21
	*luna*Δ*2*	36.4%	44
	*luna*Δ*2*	64.3%	28
	*luna*Δ*2*	64.0%	50
	*luna*Δ*2*	90.0%	40
	*luna*Δ*2*	42.9%	28
	*luna*Δ*2*	70.8%	24
	*luna*Δ*2*	59.5%	37
**Germline clone derived**	**Control**		
	*arm-lacZ/wildtype*	0.0%	303
	*arm-lacZ/wildtype*	20.9%	91
	*arm-lacZ/wildtype*	0.0%	33
	*arm-lacZ/wildtype*	0.0%	43
	*arm-lacZ/wildtype*	0.0%	190
	*arm-lacZ/wildtype*	0.0%	7
***nos::VP16-GAL4>UAS-luna-IR***	**IR**		
	*1*	67.2%	58
	*1*	81.8%	132
	*2*	97.6%	41
	*2*	81.4%	59
***nos::VP16-GAL4>UAS-white-IR***	**Control**		
	*white*	0%	500
	*white*	0%	194
	*white*	0%	170
	*white*	0%	585

Summary of defects quantified from different experimental approaches (first column). “% DNA segregation defect/bridges” represents nuclear figures during division stages, where DNA bridges remain between adjacent nuclei. Note that in the mutant scenarios generally between 50–100% of nuclear figures show bridges, whereas in the control germline clones or RNAi experiment it is mostly at 0%. These phenotypes can also be observed in early division cycles.

**Table 4:** *luna* mutant embryos show division cycle asynchrony and increased nuclear fall out defects.

Heterozygous mother derived	Maternal genotype	Asynchrony							Nuclear fall out
		stage1	stage2	stage3	% stage1	% stage2	% stage3	n total	n = centrosomes without DNA/areas
	*Df(2R)ED2155*	prophase/bridges*	-	-	100	-	-	135	17 centrosomes/4 areas
	*Df(2R)ED2155*	metaphase	anaphase	telophase	23	11	66	379	16 centrosomes/4 areas
	*Df(2R)ED2155*	telophase	interphase	bridges*	16	67	17	555	10 centrosomes/4 areas
	*luna*Δ*2*	metaphase	anaphase	telophase	34	41	25	123	0/0
	*luna*Δ*2*	metaphase	anaphase	telophase	42	45	13	135	4 centrosomes/1 area
	*luna*Δ*2*	prophase	metaphase	-	39	61	-	352	0/0
**Germline clone derived**	**Allele**								
	*luna*Δ*2*	metaphase	anaphase	telophase	22	41	37	51	0/0
	*luna*Δ*2*	metaphase	anaphase	-	32	68	-	118	0/0
	*luna*Δ*2*	metaphase	anaphase	-	46	54	-	74	0/0
	*luna*Δ*2*	metaphase	anaphase	telophase	10	29	62	21	0/0
**Germline clone derived**	**Control** **								
	*arm-lacZ/wildtype*	anaphase	metaphase	-	98	2	-	44	0/0
	*arm-lacZ/wildtype*	anaphase	metaphase	-	96	4	-	74	0/0
	*arm-lacZ/wildtype*	prophase	-	-	100	-	-	274	0/0
	*arm-lacZ/wildtype*	metaphase	-	-	100	-	-	177	7 centrosomes/2 areas
***nos::VP16-GAL4>UAS-luna-IR***	**IR**								
	*luna-RNAi2*	metaphase	interphase	-	99	1	-	267	43 centrosomes/8 areas
	*luna-RNAi2*	metaphase	interphase	-	97	3	-	158	27 centrosomes/3 areas
	*luna-RNAi1*	metaphase	anaphase	telophase	12	14	74	42	0/0
	*luna-RNAi1*	-	anaphase	telophase	-	16	84	37	0/0
***nos::VP16-GAL4>UAS-white-IR***	**Control** **								
	*white*	metaphase	-	-	100	-	-	500	8 centrosomes/1 area
	*white*	prophase	-	-	100	-	-	194	10 centrosomes/2 areas
	*white*	telophase	anaphase	-	0.82	0.18	-	170	0/0
	*white*	prophase	-	-	100	-	-	585	12 centrosomes/2 areas

Summary of defects quantified from 3 different experimental approaches (first column). Division stage asynchrony lists the different stages (1–3) and the percentage of nuclear figures found for every stage. Nuclear fallout lists how many individual centrosomes were found without associated DNA and how many such areas occurred in one embryo.

*embryo with DNA segregation defects/bridges.

**note that asynchrony is very rare/almost non-existent in control germline clone or control RNAi cohort (96–100% synchronous).

**Figure 4:**
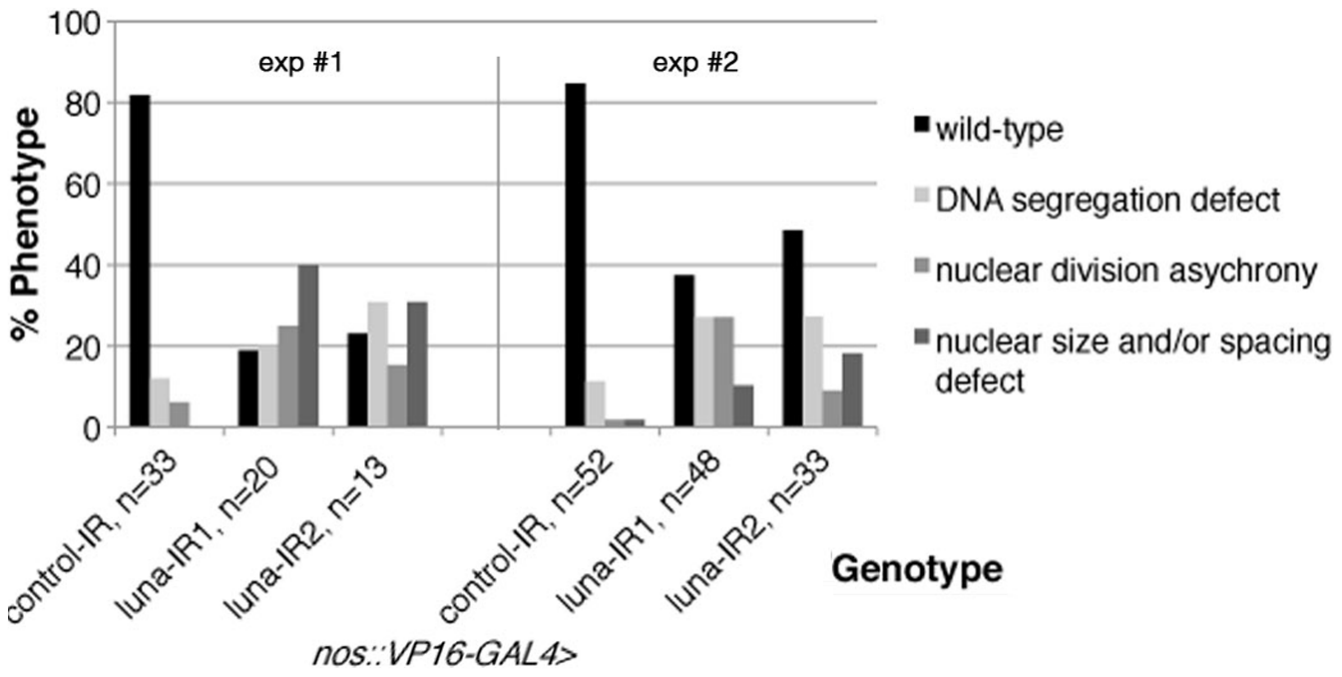
DNA segregation and division asynchrony are the most prominent *luna* phenotypes in RNAi knock-down experiments. Graph of embryonic phenotype evaluation of 2 independent maternal *luna* RNA interference experiments for 2 independent RNA sequences and control (*white* RNAi). n = number of embryos analyzed for each genotype/experiment. Nuclear fall out was not quantified in this assay.

To confirm the maternal requirement and phenotypic features, we used an independent loss-of-function approach, RNA interference with 2 non-overlapping *luna* sequences expressed via the maternal *nanos::VP16-GAL4* driver, active in the unfertilized egg [17]. In both experiments we observed similar phenotypes as mentioned above, whereas control embryos did not show such drastic effects (Figure 4).

To summarize, loss-of-function phenotypes of *luna* are apparent in mutant preblastoderm embryos derived from heterozygous mothers (Figure 2, Tables 3 and 4), homozygous mutant eggs generated by germline clone technology [16] (see below; Figure 3, Tables 3 and 4) and maternally expressed RNA interference experiments (Figure 4, Tables 3 and 4). We therefore conclude that maternal Luna function is required for embryonic development prior to cellularization and during early gastrulation of the *Drosophila* embryo and that zygotic loss of *luna* function might compound the maternal phenotype.

### *luna* is required for the coordination of DNA and centrosome replication cycles

During the early nuclear divisions in the syncytial preblastoderm embryo, prior to cellularization, centrioles are essential [18], whereas later embryogenesis and imaginal disc development can proceed normally in the absence of centrosomes [19]. During the syncytial nuclear division stages the DNA replication and centrosomal cycles can be uncoupled by reduction of the cell cycle regulators Cyclin A, B and B3 [20] or by inhibition of DNA replication [21].

When we examined *luna* loss-of-function animals derived from 3 independent approaches (see above), abnormal division patterns were observed in the pre-blastoderm embryos, at the stage when rapid, synchronized nuclear divisions take place in the syncytium (Figure 2A, D and Figure 3D). Strikingly, the most frequent phenotypic defects observed were non-segregated DNA or DNA “bridges” that remained between chromatin after the completion of nuclear divisions (Figure 2B, C and Figure 3A, B, Table 4): Such structures contained a thick DNA bridge connecting 2 prophase-like nuclei, each associated with two separated centrosomes, which appear already primed for the next cycle of nuclear division (Figures 2B and 3A, B: yellow arrowheads highlight examples of DNA bridges and white arrowheads the associated centrosomes). On average 50–100% of nuclear figures from one mutant embryo showed such DNA bridges between 2 nuclei, whereas control wild-type embryos showed generally no such defects (Figure 2B and Figures 3A, B, Table 3). In maternal RNAi interference experiments for *luna*, where all embryos with peripheal nuclei were assessed from a 2 hour collection, about 20% showed these DNA segregation defects (Figure 4). Second, asynchrony of division cycles was the other most penetrant phenotype observed (Figures 1E, 3C, and 4, and Table 4). In many cases nuclei of 3 consecutive division stages could be seen intermixed throughout the embryo. In controls, at most 2 division stages could be found and a low % of nuclei were delayed or advanced, as compared to the majority of divisions (Table 4). In addition to these prominent defects, *luna* mutant embryos displayed an increase in the nuclear fall-out phenotype as compared to control embryos (Figure 3B, C and Table 4). In several cases the remaining DNA had a fully condensed appearance and was phospho histone H3 positive, comparable to its appearance during cell cycle arrest (Figure 2E).

These defects were observed in mutant embryos derived from heterozygous mothers of *luna^Δ1^* or *luna^Δ2^* alleles (Figure 2C, E and Tables 3 and 4), *luna* deficiencies (Figure 2B, Tables 3 and 4), from germline clones for both *luna* alleles (Figure 3A–C, Tables 3 and 4), and from maternally expressed *luna* RNA interference (Figure 4, Tables 3 and 4).

### *luna* has no requirement during later developmental processes

*luna* is required for the earliest stages of development, precluding a simple analysis of larval and adult stages. However, as it is ubiquitously expressed throughout all developmental stages (flybase 2013) and in the developing eye [4], we generated homozygous mutant clones of the *luna* alleles in order to determine, if there is a requirement at later stages in development, for example during imaginal disc patterning and/or proliferation of imaginal disc cells. Surprisingly, no defects were detected in *luna* mutant tissue in the developing eye and wing, and such mutant clones had a fully wild-type appearance. Specific analyses of cell division or epithelial morphogenesis in imaginal discs, as assayed by the metaphase marker phospho histone H3 or cellular junctional integrity and associated cell adhesion (with anti-DE-cadherin) did not reveal any detectable abnormalities (Figure S1A, B), nor did we observe any defects in adult eye tissue (Figure S1C).

We conclude that *luna* is solely required for the early nuclear division cycles in the syncytial pre-blastoderm embryo and, specifically there, for the coordination of DNA replication and centrosome cycles.

### Luna and KLF6 over-expression can affect many developmental processes

Luna over-expression has been shown to interfere with normal development at larval stages and to disrupt eye development generally [4]. We wanted to test which specific processes or signaling pathways Luna could interfere with, and targeted Luna and KLF6 over-expression to subsets of cells throughout the *Drosophila* body during various stages of development, in particular to wing cells and to all head/eye cells or a subset of photoreceptor cells (Figure S2B–K). Based on Western blot analyses, expression levels were increased several fold as compared to endogenous Luna levels (Figure S2G). In all cases analyzed, normal development was severely compromised, even though *luna* is not essential at these stages and in these processes, as shown by loss-of-function analyses in the developing eye and wing and the adult eye (Figure S1). For example, in the developing eye, we find that excess levels of Luna and hKLF6 interfere with planar cell polarity establishment (Figure S2D–F) and in the wing over-expression also affects several developmental processes (Figure S2I–K). In conclusion ectopic *luna* and *hKLF6* expression cause similar defects in eye and wing development.

## Discussion

Here we describe the isolation and characterization of *luna* loss-of-function mutants in *Drosophila*. We show that mutant animals die at early embryonic stages likely due to nuclear division defects due to non-segregation of DNA at the syncytial stage, prior to cellularization and before the start of gastrulation. The *Drosophila* phenotype is reminiscent of the mouse *Klf6^−/−^* defects, where under-proliferation of hematopoietic cells in the yolk sac is the cause of early lethality [7]. Also, D'Astolfo et al. show that KLF6 is a positive regulator of cell cycle progression, and an anti-apoptotic factor, by silencing of KLF6 via siRNA in several cultured cell lines [22]. These data are consistent with the *Drosophila* loss-of-function effects in early embryos, where we observe arrested division cycles. Moreover, Racca et al describe human KLF6 localization in the syncytium of the human trophoblast in cell culture [23], another example where KLF6 is active in a syncytial tissue, like in the early *Drosophila* embryo.

*luna* expression has been shown to be maternally loaded into the egg (flybase 2007, Dmel Release 5.2 and flybase 2013). Since all early regulators of these division cycles stem from maternal contribution [9], [10], [11], the timing of the *luna* requirement and phenotype is consistent with that. We show that *luna* function is required for the synchronization of DNA and centrosome replication, starting in the embryonic syncytium. The most frequent phenotypic defects range from non segregation of DNA, thick DNA connections between 2 prophase-like structures, which are associated each with two centrosomes that are already primed to initiate the next cycle of nuclear division; Figure 2B, 3A, B) to asynchronous division cycle figures (Figure 2E, 3C), and, more rarely, trespassing mitotic spindles (Figure 2C) and nuclear fall out (Figure 3B, C). In particular, the fused DNA structures associated with 4 centrosomes seem to indicate that DNA replication or segregation is failing, while the centrosomes are ready for the next division cycle in *luna* mutant embryos. These defects were readily visible in mutant embryos from heterozygous mothers, in homozygous mutant eggs, generated via germline clones in the ovary, and in 20–30% of maternal RNAi treated embryos (Tables 3 and 4, Figure 3).

As *luna* mutant alleles manifest themselves as “early zygotic lethal like”, we generated tissue patches mutant for *luna* in developing eyes and wings. Surprisingly, such mutant clones did not display any defects in cell cycle rate or junctional/adhesion property, as assayed by the respective markers (phosphorylated histone H3 and DE-cadherin staining). Moreover, mutant adult eye clones did not reveal any phenotypic defects in cell morphogenesis, cell fate, or cellular patterning (Figure S2). These data indicate that *luna* function is dispensable or redundant for later zygotic development.

Since KLF6 is thought to be a tumor suppressor, we used several established cancerogenic and tumorigenic fly models to test if *luna* was able to ameliorate or modulate these effects. *Eyeful*, an eye over-growth scenario forming ectopic eye tissue in any location of the fly [24], and Notch signaling induced overproliferation of pluripotent eye tissue [25] were not modifiable by *luna* (data not shown).

Expression levels were checked in over-expression experiments in developing eye tissue by Western analysis. An antibody against human KLF6 recognized both over-expressed human KLF6 and *Drosophila* Luna protein, in addition to endogenous Luna on blots. In developing *Drosophila* tissue however, endogenous Luna protein was not detectable by these means, nor could we confirm by Western analysis that Luna levels were reduced in embryos from *luna* heterozygous mothers (data not shown). Generally, the expression levels of the KLF's need to be tightly regulated, as over-expression of both Luna and hKlf6 interferes with normal development [4] and our studies (Figure S2). This is not unexpected, as KLFs are known transcriptional activators (or repressors) and increasing their levels is likely to interfere with various downstream transcriptional programs and targets. According to our Western blot analysis, *Drosophila* Luna over-expression was several times the endogenous level. Similarly, misexpression/over-expression of other Klf family members in the fly eye, e.g. the founding member of this transcription factor family Krüppel itself, causes rough eyes and mis-specified photoreceptor cells [26], [27].

How do the *luna* loss-of-function defects relate to the phenotypes of other mutants/genes acting at that stage? The heterozygous maternal effect observed in *luna* was also reported for the epigenetic regulators of the Polycomb group (PcG) of genes, e.g. *polyhomeotic (ph)*, *Additional sex combs (Asc), Posterior sex combs (Psc)* and *Polycomb (Pc)* itself [28] and *polo*, *scant* double mutants [29] with both sets of genes also affecting the early syncytial division cycles. Whereas embryos from heterozygous mothers of *polo*, *scant* double mutants cause a wider array of phenotypes, mutations in the Polycomb group genes look identical to *luna* loss of function. We therefore tested whether PcG heterozygous embryos displayed changes in Luna protein levels, but no such changes were detectable (data not shown).

Several other genes show similar phenotypes, including mutations in Non-muscle Myosin/*spaghetti squash* [30] and *xpd* [31], but these show full maternal requirement for the early nuclear division cycles. However, the uncoupling of DNA replication and centrosome duplication as observed in *luna* has been described for *microcephalin* (MCPH1) [32], except that in MCPH1 mutants centrosomes were also observed to detach leading to monopolar, multipolar or acentrosomal spindles, an effect not seen in *luna* mutants. Taken together, *luna* might affect DNA status, which then leads to the secondary effect of uncoupled centrosome cycles. If *luna* were to affect the centrosomal structure alone, the DNA segregation defects should not be seen, as such phenotypes are not reported for genes essential for integral centrosome function such as *centrosomin* [33], [34], [35]. Nevertheless, the fact that *luna* is only required during the syncytial stages and not later in development indicates that the DNA segregation defect is linked to the centrosomes, since centrosomes are dispensable for later cell divisions [18], [19].

Both phenotypes, DNA segregation defects and asynchronous divisions, occur most frequently in *luna*. We speculate that the formation of DNA bridges is the primary defect and asynchronous divisions arise from unresolved and therefore delayed divisions. Similalry, nuclear fall out, a response to improperly segregated DNA could be a secondary effect [36].

*luna* mutations do not fully present themselves as dominant female sterile, as stocks can be propagated over a balancer (Table 1) and have 37–50% lethal offspring, with 8–20% lethal at preblastoderm stages (Table 2). *Poly comb* group mutations in *Pc*, *Psc* and *ph* manifest a similar effect [28].

Many experimental approaches to better understand *luna* function are precluded because of the syncytial “zygotic lethality behaviour” of *luna* loss-of-function mutants, likely a compound effect of maternal and zygotic requirements. Further studies at the syncytial blastoderm stage of embryogenesis should be possible via the recently published technique of live imaging of *in vitro* explants [37] and these could provide insight on the precise connection between *luna* loss-of-function and the early processes of syncytial nuclear divisions.

## Materials and Methods

### Fly stocks and crosses

PiggyBac elements PBac{WH}f07504, PBac{WH}f04294 and PBac{WH}f04876 were used to generate precise deletions at the *luna* locus (Figure 1), according to Parks et al., 2004 [12]. Lethal excision events were selected by complementation analysis to *Df(2R)Exel6059*, which removes approximately 24 genes. Also *Df(2R)ED2155*, which removes approximately 89 genes, was used in embryo stainings and complementation analyses in trans-heterozygous combinations.

Embryonic lethality stage was determined by comparing total number of eggs and % of lethal embryos after 2 days from balanced *luna* deletion stocks. Lethal embryos were mounted in mineral oil after dechorionation and examined for developmental stage. 2 classes of dead embryos were found: (1) unpatterned, non gastrulated and (2) segmented, cuticle containing ones. Homozygous *CyO* embryos were found to be late embryonic to first instar lethal [13].

Clones of homozygous mutant *luna* tissue were generated during later stages of development via the MARCM system.

Germline clones were induced in *hsFLP; FRT42D luna-/FRT42D ovoD* or *hsFLP; FRT42D arm-lacZ/FRT42D ovoD* females by heat shock for 1 hour at 37°C for 3 consecutive days from first larval instar stage on. Such females were crossed to *w^1118^* males and all eggs collected in 7–18 hrs intervals and fixed until females stopped laying. Standard embryo fixation and antibody staining was performed according to Cooley lab protocols. All crosses were performed at 25°C.

For RNA interference during oogenesis freshly eclosed *nanos::VP16-GAL4/+; UAS-luna-IR/+* females were crossed to *w^1118^* males at 29°C for 4 days in well yeasted bottles. Embryos were collected in 2 hour intervals at 29°C and stained as described above. For 2 collections epifluorescence pictures of Hoechst staining were taken, representative of all embryos, where nuclei had reached the cortex. These pictures were categorized according to nuclear division stages and defects for Figure 4. Control embryos were *nanos::VP16-GAL4/+; UAS-white-IR/+*.

For over-expression, *UAS-luna[DG]*, *luna-EP* insertions, UAS-hKLF6 and GAL4 drivers *sevenless* (*sev*-GAL4), *engrailed* (*en-GAL4*) and *scalloped* (*sd*-GAL4) were used.

### Imaging and histology

Embryos were collected from *luna^−^/CyO* stocks or *luna^−^/CyO* and *Df(2R)Exel6059/CyO* or *Df(2R)ED2155* intercrosses, germline clone crosses (see above) or from *nanos::VP16-GAL4/+, UAS-luna-RNAi-1* or *-2/+* mothers at 29°C and stained for DNA with Hoechst, phosphorylated histone H3, gamma-tubulin or centrosomin, and cylin B.

Confocal microscopy was performed on a Zeiss Meta LSM 510. Images are projections of several consecutive grazing sections.

Antibodies and dilutions used:

rat anti-Elav (1∶50 from Developmental Studies Hybridoma Bank/DSHB),
rabbit anti-phospho histone H3 (1∶200, Upstate Biotechnology),
rat anti-DEcad (1∶20 from DSHB),
rabbit anti-Cnn (1∶100, kindly provided by Tom Kaufmann),
mouse anti-gamma tubulin (1∶500 from Sigma),
mouse anti-cycB (1∶5 from DSHB),
Hoechst 33342 (1∶500 from Sigma)
rabbit anti-hKLF6 (1∶400 for tissue and 1∶1000 for Western from Santa Cruz).

Phenotypic analysis was based on confocal images covering half to 2/3 of the embryo cortex and ImageJ was used to count division figures and organelles for Tables 3 and 4.

### Constructs and molecular analysis

UAS-KLF6 was cloned by PCR, amplifying the KLF6 coding sequence with forward primer containing 5′ atggacgtgctccccatgtgc 3′ sequences and reverse primer 5′ tcagaggtgcctcttcatgtg 3′. The resulting PCR product was cloned as EcoRI fragment into the pUAST vector. The final construct was confirmed by DNA sequencing in both orientations.

UAS-lunaRNAi constructs were generated as previously described [38]. Primer sets were designed using the Heidelberg eRNAi prediction site (www.dkfz.de/signaling2/e-rnai).

UAS-lunaRNAi-2: FWD-RNAi-31071 5′ CCTAGGACGAGTAGTAGCCGGTGGTG 3′ and REV-RNAi-31071 5′ GGATCCATCGCGAGTGCTAAAATGCT-3′),

UAS-lunaRNAi-1: FWD-RNAi-31812 5′ CCTAGGACCTGTTGCCATTGATCCTC 3′ and REV-RNAi-31812 5′ GGATCCTTGCATCAAAAGCCAACTCA 3′.

Flanking AvrII and BamHI restriction sites were added (underlined). PCR amplified sequences were cloned via the DNA topoisomerase I technique. Constructs were sequenced in both orientations.

Western and *in vivo* Klf6/Luna stainings: Anti-human Klf6 was used to probe d.m. Luna and human KLF6 over-expression in *Drosophila* eye discs tissue and on Western blots thereof. The equivalent of 40 eye discs of each genotype was loaded on a gel. Eye disc staining of the same genotypes revealed only a Klf6 specific pattern. Human tissue culture cells: Blot detection was performed using standard HRP coupled secondary antibodies and ECL detection according to protocol.

## Supporting Information

Figure S1 ***luna*** **mutant tissue in developing eyes and wings and in adult eyes, do not show defects.** Anterior is to the left and dorsal is up in all panels. 3rd larval instar eye (A) and wing (B) imaginal disc tissue mutant for *luna*Δ*1#b5* or *#c7*, respectively (marked by GFP in green), stained for DE-cadherin (red and monochrome in A′, B′) and metaphase (phospho-histone H3 in blue, monochrome in A″, B″). (C) Adult eye section of *luna*Δ*1#c10* loss-of-function clone marked by the loss of pigment granules next to rhabdomeres. (C′) Schematic representation of mutant eye tissue in grey.
(TIF)

Figure S2 **Luna and KLF6 overexpression affect eye and wing development.** A–C: Lateral and dorsal views of adult heads, D–F: Tangential eye sections and schematic presentations (D′–F′) indicating planar cell polarity (PCP) defects. Anterior is to the left and dorsal up. Black and red arrows represent the two chiral forms of ommmatidia, green arrows represent ommatidia, which have a symmetric rhabdomere/photoreceptor arrangement. Circles indicate ommatidia with loss of photoreceptors. G: Western blot of Luna/KLF6 of over expression. H–K: Adult wings, anterior is up and proximal to the left. (A) Wild-type eye. (B) *UAS-Luna[EY08b]/CyO; sev-GAL4[K25]* at 29°C shows a small, rough eye. (C) *eyFLP3.5/+; UAS-Luna[DeGraeve]/+; act>y+>GAL4/+* at 18°C shows severely affected head and eye structures. (D, D′) *UAS-Luna[EY08b]/+; sev-GAL4[K25]* at 29°C eyes display misrotated ommatidia and chirality defects. (E, E′) *UAS-Luna [DG]/+, sev-GAL4[K25]/+* at 25°C and (F, F′) *UAS-KLF6 (#7.1)/+, sev-GAL4[K25]/+* at 29°C show similar PCP defects. (G) Anti-human KLF6 antibody detects Luna on Western blots. Protein blot of human cell lines BPH, transfected with KLF6 and untransfected PC3M cells as controls, endogenous Luna levels (*UAS-KLF6*, *UAS-luna* and *sev-GAL4*), over expressed KLF6 and Luna levels in *Drosophila* eye imaginal discs probed for KLF6 and actin as loading control. *UAS-luna* is several fold over-expressed compared to endogenous levels; compare 4 right lanes (overexpressed) vs. the adjacent 3 left lanes (endogenous). (H) Wild-type wing of *sd-GAL4*/Y; *Sb*/+ genotype at 18°C as control. (I–K): KLF6 and Luna over-expression in the wing cause loss of margin pattern (I, J), vein defects, ectopic bristles (J, K) and decrease of wing hair density (K). Genotypes and temperature: (I) *sd-GAL4/Y; UAS-KLF6/+* at 18°C. (J) *sd-GAL4/+; UAS-Luna[DG]/+* at 16°C. (K) *en-GAL4/+ UAS-KLF6/+* at 29°C.
(TIF)

## References

[1] McConnell BB, Yang VW (2010) Mammalian Kruppel-like factors in health and diseases. Physiol Rev 90: 1337–1381. 20959618 10.1152/physrev.00058.2009 PMC2975554

[2] Ema M, Mori D, Niwa H, Hasegawa Y, Yamanaka Y, et al (2008) Kruppel-like factor 5 is essential for blastocyst development and the normal self-renewal of mouse ESCs. Cell Stem Cell 3: 555–567. 18983969 10.1016/j.stem.2008.09.003

[3] Kaczynski J, Cook T, Urrutia R (2003) Sp1- and Kruppel-like transcription factors. Genome Biol 4: 206. 12620113 10.1186/gb-2003-4-2-206 PMC151296

[4] De Graeve F, Smaldone S, Laub F, Mlodzik M, Bhat M, et al (2003) Identification of the Drosophila progenitor of mammalian Kruppel-like factors 6 and 7 and a determinant of fly development. Gene 314: 55–62. 14527717 10.1016/s0378-1119(03)00720-0

[5] Seetharam A, Bai Y, Stuart GW (2010) A survey of well conserved families of C2H2 zinc-finger genes in Daphnia. BMC Genomics 11: 276. 20433734 10.1186/1471-2164-11-276 PMC2889900

[6] Laub F, Lei L, Sumiyoshi H, Kajimura D, Dragomir C, et al (2005) Transcription factor KLF7 is important for neuronal morphogenesis in selected regions of the nervous system. Mol Cell Biol 25: 5699–5711. 15964824 10.1128/MCB.25.13.5699-5711.2005 PMC1157008

[7] Matsumoto N, Kubo A, Liu H, Akita K, Laub F, et al (2006) Developmental regulation of yolk sac hematopoiesis by Kruppel-like factor 6. Blood 107: 1357–1365. 16234353 10.1182/blood-2005-05-1916 PMC1895396

[8] Zhao X, Monson C, Gao C, Gouon-Evans V, Matsumoto N, et al (2010) Klf6/copeb is required for hepatic outgrowth in zebrafish and for hepatocyte specification in mouse ES cells. Dev Biol 344: 79–93. 20430021 10.1016/j.ydbio.2010.04.018 PMC2909330

[9] Murray AWK, Kirschner MW (1991) What controls the cell cycle? Sci Am 264 (3) 56–63. 1828616 10.1038/scientificamerican0391-56

[10] Foe VE, Odell GM, Edgar BA (1993) Mitosis and morphogenesis in the Drosophila embryo: Point and counterpoint. In: The Development of Drosophila melanogaster, MBate and AMartinez Arias, eds. Cold Spring Harbor Laboratory Press, pp. 149–300.

[11] Edgar BA, Schubiger G (1986) Parameters controlling transcriptional activation during early Drosophila development. Cell 44: 871–877. 2420468 10.1016/0092-8674(86)90009-7

[12] Parks AL, Cook KR, Belvin M, Dompe NA, Fawcett R, et al (2004) Systematic generation of high-resolution deletion coverage of the Drosophila melanogaster genome. Nat Genet 36: 288–292. 14981519 10.1038/ng1312

[13] Simpson P (1983) Maternal-Zygotic Gene Interactions during Formation of the Dorsoventral Pattern in Drosophila Embryos. Genetics 105: 615–632. 17246169 10.1093/genetics/105.3.615 PMC1202177

[14] Edgar BA, Kiehle CP, Schubiger G (1986) Cell cycle control by the nucleo-cytoplasmic ratio in early Drosophila development. Cell 44: 365–372. 3080248 10.1016/0092-8674(86)90771-3

[15] De Renzis S, Elemento O, Tavazoie S, Wieschaus EF (2007) Unmasking activation of the zygotic genome using chromosomal deletions in the Drosophila embryo. PLoS Biol 5: e117. 17456005 10.1371/journal.pbio.0050117 PMC1854917

[16] Chou TB, Noll E, Perrimon N (1993) Autosomal P[ovoD1] dominant female-sterile insertions in Drosophila and their use in generating germ-line chimeras. Development 119: 1359–1369. 8306893 10.1242/dev.119.4.1359

[17] Van Doren M, Williamson AL, Lehmann R (1998) Regulation of zygotic gene expression in Drosophila primordial germ cells. Curr Biol 8: 243–246. 9501989 10.1016/s0960-9822(98)70091-0

[18] Rodrigues-Martins A, Riparbelli M, Callaini G, Glover DM, Bettencourt-Dias M (2008) From centriole biogenesis to cellular function: centrioles are essential for cell division at critical developmental stages. Cell Cycle 7: 11–16. 18196975 10.4161/cc.7.1.5226

[19] Basto R, Lau J, Vinogradova T, Gardiol A, Woods CG, et al (2006) Flies without centrioles. Cell 125: 1375–1386. 16814722 10.1016/j.cell.2006.05.025

[20] McCleland ML, O'Farrell PH (2008) RNAi of mitotic cyclins in Drosophila uncouples the nuclear and centrosome cycle. Curr Biol 18: 245–254. 18291653 10.1016/j.cub.2008.01.041 PMC2698964

[21] Raff JW, Glover DM (1988) Nuclear and cytoplasmic mitotic cycles continue in Drosophila embryos in which DNA synthesis is inhibited with aphidicolin. J Cell Biol 107: 2009–2019. 3143733 10.1083/jcb.107.6.2009 PMC2115639

[22] D'Astolfo DS, Gehrau RC, Bocco JL, Koritschoner NP (2008) Silencing of the transcription factor KLF6 by siRNA leads to cell cycle arrest and sensitizes cells to apoptosis induced by DNA damage. Cell Death Differ 15: 613–616. 18188167 10.1038/sj.cdd.4402299

[23] Racca AC, Camolotto SA, Ridano ME, Bocco JL, Genti-Raimondi S, et al (2011) Kruppel-like factor 6 expression changes during trophoblast syncytialization and transactivates sshCG and PSG placental genes. PLoS One 6: e22438. 21799854 10.1371/journal.pone.0022438 PMC3142166

[24] Ferres-Marco D, Gutierrez-Garcia I, Vallejo DM, Bolivar J, Gutierrez-Avino FJ, et al (2006) Epigenetic silencers and Notch collaborate to promote malignant tumours by Rb silencing. Nature 439: 430–436. 16437107 10.1038/nature04376

[25] Reynolds-Kenneally J, Mlodzik M (2005) Notch signaling controls proliferation through cell-autonomous and non-autonomous mechanisms in the Drosophila eye. Dev Biol 285: 38–48. 16039641 10.1016/j.ydbio.2005.05.038

[26] Preiss A, Rosenberg UB, Kienlin A, Seifert E, Jackle H (1985) Molecular genetics of Kruppel, a gene required for segmentation of the Drosophila embryo. Nature 313: 27–32. 3917552 10.1038/313027a0

[27] Carrera P, Abrell S, Kerber B, Walldorf U, Preiss A, et al (1998) A modifier screen in the eye reveals control genes for Kruppel activity in the Drosophila embryo. Proc Natl Acad Sci U S A 95: 10779–10784. 9724781 10.1073/pnas.95.18.10779 PMC27972

[28] O'Dor E, Beck SA, Brock HW (2006) Polycomb group mutants exhibit mitotic defects in syncytial cell cycles of Drosophila embryos. Dev Biol 290: 312–322. 16388795 10.1016/j.ydbio.2005.11.015

[29] Archambault V, Zhao X, White-Cooper H, Carpenter AT, Glover DM (2007) Mutations in Drosophila Greatwall/Scant reveal its roles in mitosis and meiosis and interdependence with Polo kinase. PLoS Genet 3: e200. 17997611 10.1371/journal.pgen.0030200 PMC2065886

[30] Royou A, Field C, Sisson JC, Sullivan W, Karess R (2004) Reassessing the role and dynamics of nonmuscle myosin II during furrow formation in early Drosophila embryos. Mol Biol Cell 15: 838–850. 14657248 10.1091/mbc.E03-06-0440 PMC329397

[31] Li X, Urwyler O, Suter B (2010) Drosophila Xpd regulates Cdk7 localization, mitotic kinase activity, spindle dynamics, and chromosome segregation. PLoS Genet 6: e1000876. 20300654 10.1371/journal.pgen.1000876 PMC2837399

[32] Brunk K, Vernay B, Griffith E, Reynolds NL, Strutt D, et al (2007) Microcephalin coordinates mitosis in the syncytial Drosophila embryo. J Cell Sci 120: 3578–3588. 17895363 10.1242/jcs.014290

[33] Megraw TL, Li K, Kao LR, Kaufman TC (1999) The centrosomin protein is required for centrosome assembly and function during cleavage in Drosophila. Development 126: 2829–2839. 10357928 10.1242/dev.126.13.2829

[34] Vaizel-Ohayon D, Schejter ED (1999) Mutations in centrosomin reveal requirements for centrosomal function during early Drosophila embryogenesis. Curr Biol 9: 889–898. 10469591 10.1016/s0960-9822(99)80393-5

[35] Zhang J, Megraw TL (2007) Proper recruitment of gamma-tubulin and D-TACC/Msps to embryonic Drosophila centrosomes requires Centrosomin Motif 1. Mol Biol Cell 18: 4037–4049. 17671162 10.1091/mbc.E07-05-0474 PMC1995719

[36] Takada S, Kelkar A, Theurkauf WE (2003) Drosophila checkpoint kinase 2 couples centrosome function and spindle assembly to genomic integrity. Cell 113: 87–99. 12679037 10.1016/s0092-8674(03)00202-2

[37] Telley IA, Gaspar I, Ephrussi A, Surrey T (2013) A single Drosophila embryo extract for the study of mitosis ex vivo. Nat Protoc 8: 310–324. 23329004 10.1038/nprot.2013.003

[38] Jenny A, Mlodzik M (2008) Modified vectors for the two-step directional cloning of inverted repeats for RNA interference in Drosophila. Biotechniques 44: 335–339. 18361787 10.2144/000112720 PMC2796131

