# Supplementary figures and images for: Luna, a Drosophila KLF6/KLF7, Is Maternally Required for Synchronized Nuclear and Centrosome Cycles in the Preblastoderm Embryo
Source: PLoS One. 2014 Jun 10;9(6):e96933. doi: 10.1371/journal.pone.0096933 (PMC4051582; doi:10.1371/journal.pone.0096933)

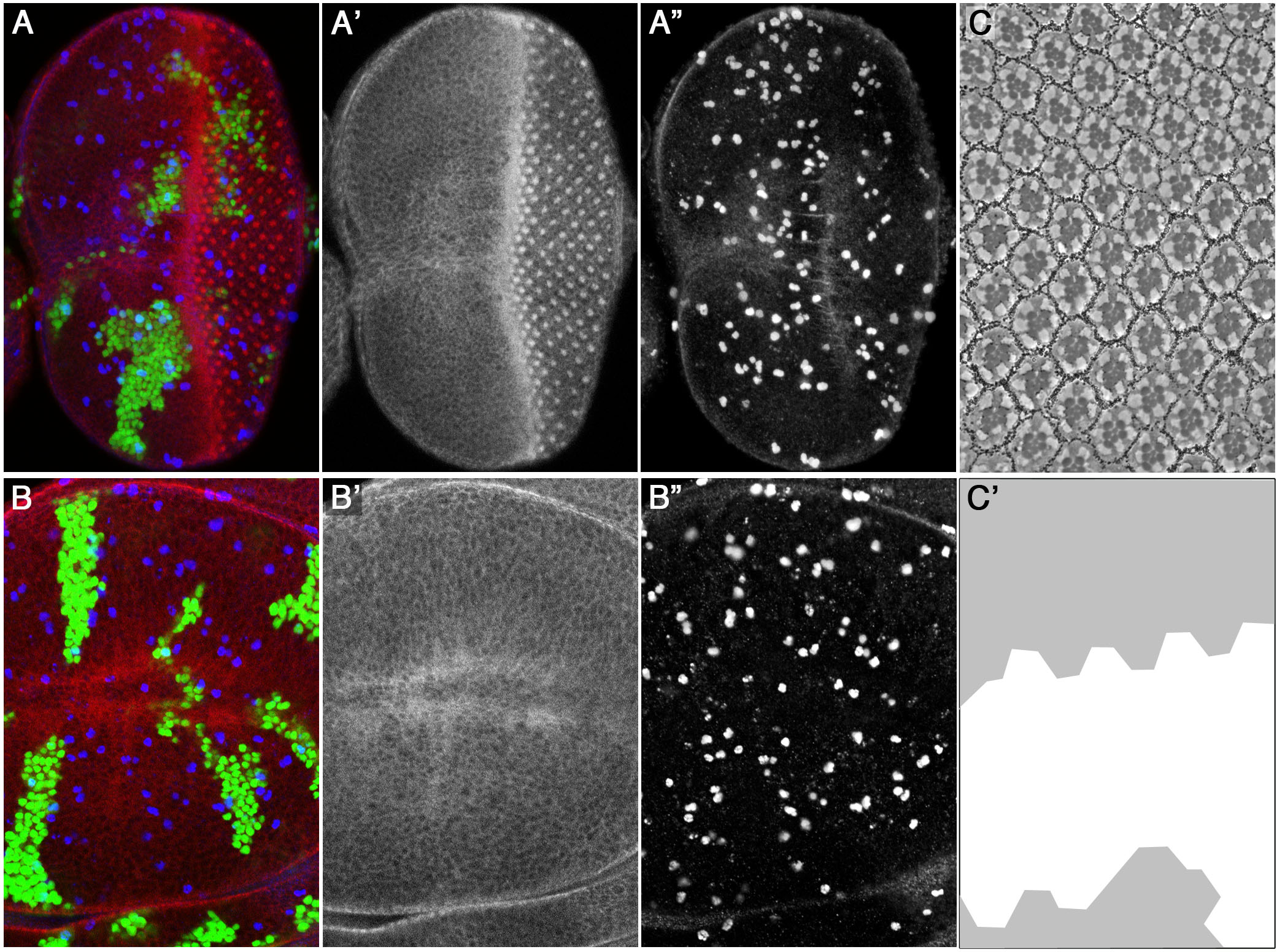

Supplement: Figure S1 — luna mutant tissue in developing eyes and wings and in adult eyes, do not show defects. Anterior is to the left and dorsal is up in all panels. 3rd larval instar eye (A) and wing (B) imaginal disc tissue mutant for lunaΔ1#b5 or #c7, respectively (marked by GFP in green), stained for DE-cadherin (red and monochrome in A′, B′) and metaphase (phospho-histone H3 in blue, monochrome in A″, B″). (C) Adult eye section of lunaΔ1#c10 loss-of-function clone marked by the loss of pigment granules next to rhabdomeres. (C′) Schematic representation of mutant eye tissue in grey. (TIF) [file pone.0096933.s001.tif]

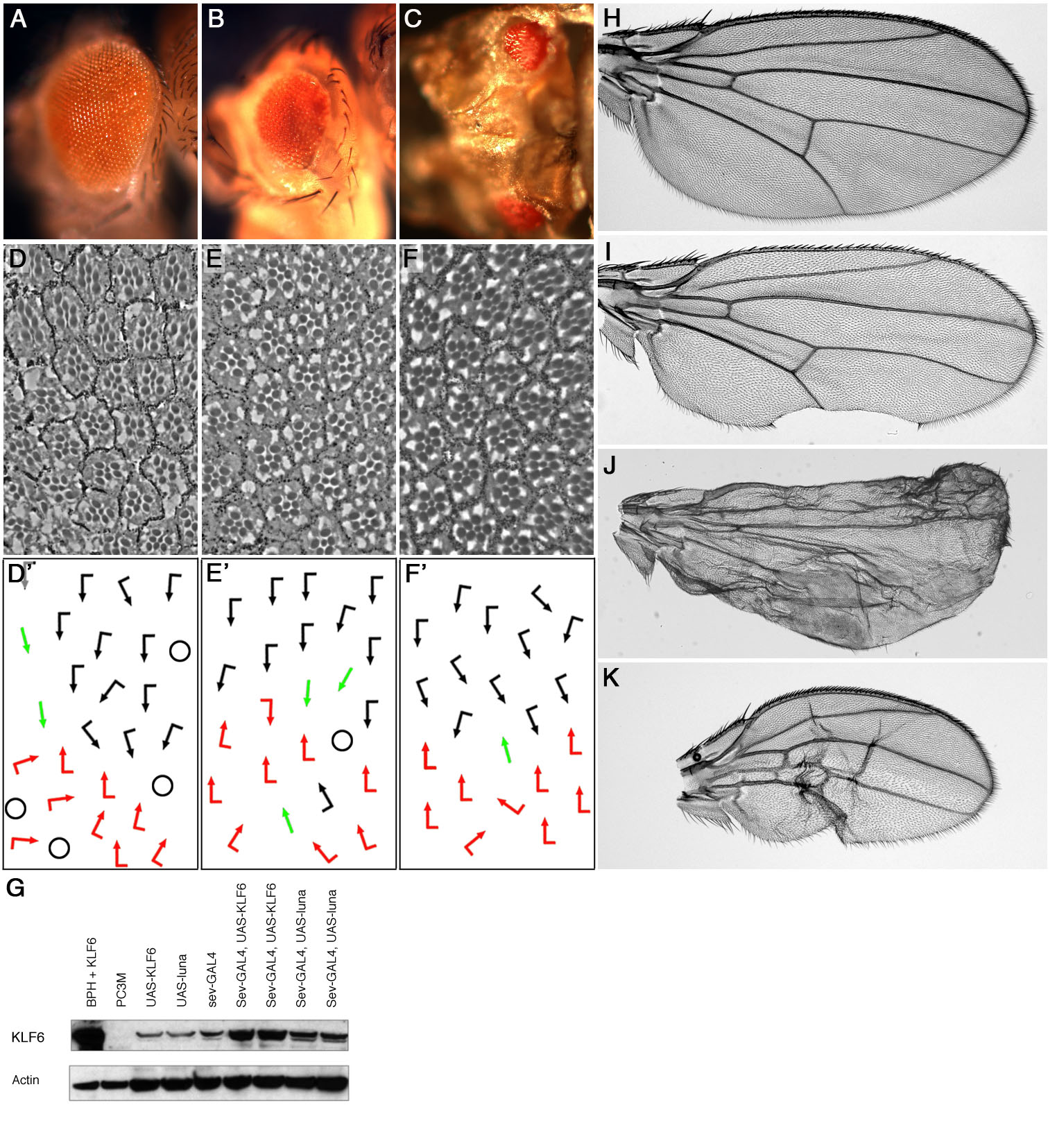

Supplement: Figure S2 — Luna and KLF6 overexpression affect eye and wing development. A–C: Lateral and dorsal views of adult heads, D–F: Tangential eye sections and schematic presentations (D′–F′) indicating planar cell polarity (PCP) defects. Anterior is to the left and dorsal up. Black and red arrows represent the two chiral forms of ommmatidia, green arrows represent ommatidia, which have a symmetric rhabdomere/photoreceptor arrangement. Circles indicate ommatidia with loss of photoreceptors. G: Western blot of Luna/KLF6 of over expression. H–K: Adult wings, anterior is up and proximal to the left. (A) Wild-type eye. (B) UAS-Luna[EY08b]/CyO; sev-GAL4[K25] at 29°C shows a small, rough eye. (C) eyFLP3.5/+; UAS-Luna[DeGraeve]/+; act>y+>GAL4/+ at 18°C shows severely affected head and eye structures. (D, D′) UAS-Luna[EY08b]/+; sev-GAL4[K25] at 29°C eyes display misrotated ommatidia and chirality defects. (E, E′) UAS-Luna [DG]/+, sev-GAL4[K25]/+ at 25°C and (F, F′) UAS-KLF6 (#7.1)/+, sev-GAL4[K25]/+ at 29°C show similar PCP defects. (G) Anti-human KLF6 antibody detects Luna on Western blots. Protein blot of human cell lines BPH, transfected with KLF6 and untransfected PC3M cells as controls, endogenous Luna levels (UAS-KLF6, UAS-luna and sev-GAL4), over expressed KLF6 and Luna levels in Drosophila eye imaginal discs probed for KLF6 and actin as loading control. UAS-luna is several fold over-expressed compared to endogenous levels; compare 4 right lanes (overexpressed) vs. the adjacent 3 left lanes (endogenous). (H) Wild-type wing of sd-GAL4/Y; Sb/+ genotype at 18°C as control. (I–K): KLF6 and Luna over-expression in the wing cause loss of margin pattern (I, J), vein defects, ectopic bristles (J, K) and decrease of wing hair density (K). Genotypes and temperature: (I) sd-GAL4/Y; UAS-KLF6/+ at 18°C. (J) sd-GAL4/+; UAS-Luna[DG]/+ at 16°C. (K) en-GAL4/+ UAS-KLF6/+ at 29°C. (TIF) [file pone.0096933.s002.tif]
